# Incidence of *Listeria* spp. in Dairy Cows Feed and Raw Milk in Latvia

**DOI:** 10.5402/2012/435187

**Published:** 2012-01-26

**Authors:** I. H. Konosonoka, A. Jemeljanovs, B. Osmane, D. Ikauniece, G. Gulbe

**Affiliations:** Scientific Institute of Biotechnology and Veterinary Medicine (SIGRA), Latvia University of Agriculture, Instituta street No. 1, 2150 Sigulda, Latvia

## Abstract

Feed is a risk factor for poisoning the farm environment thus also fresh milk with pathogenic microorganisms of *Listeria* genus species. *Listeria ivanovii*, *Listeria innocua*, and *Listeria seeligeri* were isolated from 9.2%, but *Listeria monocytogenes* from 20.0% of feed samples. Most often different fodders (9.3%) and silage (4.7%) were contaminated with *Listeria monocytogenes*. *Listeria* genus species were isolated more often from feed prepared and used in organic dairy farm than from that used in conventional dairy farm, correspondingly 44.4% and 18.3%. No *Listeria monocytogenes* was found in bulk milk samples of organic dairy farm.

## 1. Introduction

Dairy farming is the leading sector of the Latvia agriculture. We had 7 040 dairy herds with 121 945 cows in Latvia at the 2010. Output of milk is 23%, what is the greatest from all agricultural products. Milk processing has always been an important part of Latvia's national economy. Production level of milk products has been increased from 93.9 million LVL at 2002 to 218.2 million LVL at 2008 [1]. Milk and milk products are a traditional part of the Latvian diets. Enhanced nutrition qualities, taste, and health benefits have all been advocated as reasons for increase interest in raw milk consumption. There are 29 states in Europe, int. al. Latvia, which allow the sale of raw milk [2]. Unfortunately, milk is a good source of nutrients and edible energy not only for humans but also for numerous microorganisms, which thus can grow in milk. These microorganisms are primarily bacteria, but some moulds and yeasts can also grow in milk. The presence of several species of microorganisms in raw milk is undesirable, either because the organisms can be pathogenic, or because their growth results in undesirable transformations in the milk [2, 3]. Bacterial food poisoning is an illness caused by the consumption of food int. al. contaminated with bacteria or bacterial toxins. It is known that bacterial toxins may act as very danger food poisoning substances [4].

 The often listed pathogens in raw milk are *Staphylococcus aureus*, *Escherichia coli, Salmonella *spp.,* Shigella *spp.,* Yersinia enterocolitica, Aeromonas hydrophila, Brucella abortus, Campylobacter jejuni, Bacillus cereus, *and* Listeria monocytogenes *(*L. monocytogenes*), among them [3, 5, 6].

 There are six species in the genus *Listeria*—*L. monocytogenes, L. ivanovii, L. innocua, L. seeligeri, L. Welshimeri, *and* L. grayi. *Results of genomic analysis suggest that these species fall into three main groups. The first contains *L. monocytogenes, L. Innocua, *and* L. welshimeri*, the second group *L. ivanovii* and *L. seeligeri*, and the third *L. grayi* [7]. Only two, *L. monocytogenes* and *L. ivanovii*, are pathogenic for humans and animals [8, 9]. The cellular behaviour of *L. ivanovii* is quite similar to that of *L. monocytogenes*, and the virulence gene cluster of *L. monocytogenes* is present in the other pathogenic species *L. ivanovii* [10]. Investigations of Chakraborty et al. [11] reveal that the ability to grow in the host cytoplasm cause vasodilator-stimulated phosphoprotein, which is characteristic both for *L. monocytogenes* and *L. ivanovii*. *L. monocytogenes* is a high adaptable environmental bacterium capable of existing both as animal pathogen and plant saprophyte with powerful array of regulated virulence factors [12]. At the same time it is potentially lethal foodborne pathogen commonly found in dairy cows' environment—in cow feces, silage, soil, water, and so forth [13]. It is proved that *L. monocytogenes* grows into biofilms attached to the surfaces in food-processing plants [14, 15] and milking systems in dairy farms. The common treatment of surfaces is not effective to eliminate this dangerous foodborne pathogen, and it easily can pass into raw milk. *L. monocytogenes* can cause a rare but serious disease called listeriosis, especially among pregnant women, the elderly, or individuals with a weakened immune system. *L. monocytogenes* is more likely to cause death than other bacteria that cause food poisoning. 20 to 30% of foodborne listeriosis infections in high-risk individuals may be fatal [16].

 The presence of pathogens depends on ingestion of contaminated feed followed by amplification in bovine hosts and fecal dissemination in the farm environment. The final outcome of this cycle is a constantly maintained reservoir of foodborne pathogens that can reach humans by direct contact, ingestion of raw contaminated milk or cheese, or contamination during the processing of milk products [17].

 Raw milk is offered for sale in every market place in Latvia. Therefore it is essential to gather information about microbial risk factors and hazards associated with raw milk production. Risk assessment and microbial monitoring will continue to play important role in ensuring food safety [4]. Critical control point management programmes created for individual milk production farms based upon risk analysis, total quality management and hazard analysis, and critical control point principles are essential for obtaining safe and healthy raw milk for consumers and for processing.

 The objective of this study was to clarify incidence of bacteria from the genus *Listeria* int. al. foodborne pathogen *L. monocytogenes* in the feed and raw milk from one organic and three conventional dairy farms in Latvia.

## 2. Materials and Methods

### 2.1. Sampling

The research was carried out from June 2008 to May 2010. In total, 130 feed samples and 244 bulk tank milk samples from organic farm “Grantskalni” and three conventional farms “Palsa,” “Lacplesa piens,” and “Robeznieki” were analyzed. Feed and milk samples were collected randomly in all seasons of year.

### 2.2. Isolation of *Listeria* spp

*L. monocytogenes* from feed and milk samples were isolated in accordance with international standard LVS EN ISO 11290-1+A1 “Microbiology of food and animal feeding stuffs—Horizontal method for detection and enumeration of *Listeria monocytogenes*—Part 1: detection method”. Presumptive *L. monocytogenes* isolates were purified and confirmed by the Fourier transform infrared spectroscopy (FT-IR) technique.

**Identification of Listeria Species Using FT-IR**

Sample preparation and measurement of FT-IR spectra were performed according to manufacturer's instructions using an infrared spectrometer Tensor 27 and software OPUS version 6.5 (Bruker optic GmbH, Germany). The bacterial strains were subcultured on tryptone soya agar (TSA, OXOID) by incubating the plates at 37°C for 24 h. Bacteria inoculum was transferred from the TSA preculture onto the surface of the TSA plate with a loop and spread with spatula until homogeneity bacterial lawn (one half of the agar plate is enough for each strain). Prepared plates were incubated for 24 h at 30°C. After incubation suspensions with 2 full loops of the bacteria scraped from the confluent lawn in 100 *μ*L distilled water in the Eppendorf tubes were prepared. Afterwards we homogenized this suspension with a vortex for 10 sec., transferred 25 *μ*L of the suspension on the microtiter plate, and dried about 45 min. at 42°C. For the FT-IR absorption measurements we used 32 scans, 6 cm^−1^ resolution, 24 phase resolution, and repeated background position measurements between 4,000 and 500 cm^−1^. Identification of bacteria was based on Bruker's spectral library of *Listeria* species which includes reference strains of* L. monocytogenes, L. innocua, L. ivanovii, L. Welshimeri,* and *L. seeligeri*.

### 2.3. Statistical Analysis

*Listeria *spp. and *L. monocytogenes* prevalence were analyzed with Statistical Package for Social Sciences 17.0 for Windows (SPSS Inc., Chicago, IL, USA).

## 3. Results and Discussion

The bacteria of *Listeria *species were detected in feed samples (*n* = 130) in 38 cases or 29.2%. In 12 (9.2%) these were *L. innocua, L. Ivanovii,* and *L. seeligeri*, but in 26 cases (20.0%) *L. monocytogenes*.

Bacteria of* Listeria* genus can be widely found in nature—in soil, on plants, in waters, on animal hair and birds' bodies, and so forth. Thus they can easily get into the feed for dairy cows. Literature sources show that both *L. monocytogenes* and *L. ivanovii *cause animal and human infections. *L. ivanovii *shares certain characteristics with *L. monocytogenes* (e.g., hemolysis) and is occasionally associated with abortion in ruminants [9]. Therefore incidence of these two species in animal feed is a risk factor for presence of *Listeria* in the farm environment, cow infections, their presence in milk and thus also in human body causing infections.

Animal and human pathogen *L. monocytogenes* was isolated from 26 feed samples (20.0%). Milk plays important role in *L. monocytogenes* epidemiology [18, 19]; therefore it must be kept in mind that these dangerous bacteria are brought in the farm environment by contaminated feed and thus also on cow hair, udder, and teat skin and then also in milk. *L. monocytogenes *is known as cow mastitis [20], conjunctivitis, and other disease-causing pathogen microorganisms. It has been proved that *Listeria* strains isolated from infections have been found also in farm environment—in feces and silage which means that *L. monocytogenes* strains found in nature are virulent [21].

Incidence of* Listeria *spp. and* L. monocytogenes* in different type of feed has been summarized in Table 1.

As it can be seen, the data summarized in Table 1 show that none of 14 different grass samples contains bacteria of *Listeria* species. Thus, grass has not been in contact with dung, wild animal feces, which can cause the presence of *Listeria* in the soil and on plants.

Silage is considered as the main source of *L. monocytogenes* and other *Listeria* genus bacteria in farm environment [22]. However, neither silage (*n* = 48) nor haylage (*n* = 16) were highly contaminated in our study; it ranges from 1.5 to 4.7% (Table 1).

Dry food products were contaminated with *L. monocytogenes*: feed mixture prepared in the farm—1.5%, different fodder products (different corns, rape cakes, etc.)—9.3%, straw—1.5%, and hay—1.5% of all tested samples (*n* = 130) (Table 1). The tested straw and hay were stored at the field during winter therefore exposed to long-term impact of environment. Birds and wild animals are the main carriers of *Listeria* spp. in nature, so they also could contaminate straw and hay used in the farm. Hay used as fodder might have been contaminated on the field by bird and animal feces. Listerias are gram-positive bacteria with different structure and chemical content of its cell wall and thus more resistant than gram-negative ones. *Listeria* cell wall protects the inner content of the cell against impact of external mechanical and osmotic force, insufficient humidity and pH level, and other damaging growing and reproduction factors. The cell wall of gram-positive bacteria consists of peptidoglycan which is associated with teichoic acids and lipoteichoic acids in complex multilayer structure, while the cell wall of gram-negative bacteria consists of one peptidoglycan layer which is covered with a membrane [5, 23]. Thus, the cell wall of gram-positive bacteria, including *Listeria* is tenfold thicker than the cell wall of gram-negative bacteria, and they are much viable in external environment, as well as considerably resistant to disinfectants use for cow teat treatment before milking. *Listeria *spp. can multiply in diverse environmental conditions. It is able to grow at temperatures from +1°C, they can be considered as psychrophilic microorganisms [24] capable of surviving, growing, and multiplying in feed also in autumn and winter, when straw, hay roll, and hay are on the field or under open sheds.

Table 2 shows summarized obtained data on incidence of *Listeria *spp. and *L. monocytogenes* in organic and conventional farms and feed used in different seasons.

 The data summarized in Table 2 show that feed was free of *Listeria* during summer in both organic and conventional farms. Our studies are proved by literature data on the fact that *Listeria *spp. is most likely isolated from feedstuff in winter and autumn than it is in summer [25, 26]. The obtained results show that both *Listeria *spp. and *L. monocytogenes* are found more often in feed prepared in organic farm (correspondingly 14.8% and 29.6%) than in feed used in conventional farms (correspondingly 5.2% and 13.1%). Results are logical as, according to the legislative acts of the European Union, organic farms should use as less chemical substances as possible; for example, ferments, yeast, and bacteria should be used as silage additives instead of chemical preservatives thus limiting multiplication of pathogenic bacteria.

 Although different feed samples contained *L. monocytogenes*, no such bacteria were found in samples of bulk milk from organic farm (*n* = 33), but in samples of bulk milk from conventional farm *L. monocytogenes* was found three times or in 1.4% of all cases (*n* = 211). Similar results were obtained in studies of other scientists. Fernandez et al. [27] has isolated *L. monocytogenes* from 3.0% (*n* = 140), Jayarao and Henning [28] from 3.8%, but Vilar et al. [29] from 6.1% of bulk milk samples.

The obtained results show that accurate observing of hygiene standards concerning treatment of cows' udder and teats, as well as proper washing and cleaning process of milking system pipe lines and cooling tanks, protects the milk against bacteria.

## 4. Conclusions

Different type of feed is a risk factor for poisoning the farm environment thus also poisoning fresh milk with pathogenic microorganisms of *Listeria* genus species in both organic and conventional farms. *Listeria ivanovii, Listeria innocua, *and* Listeria seeligeri *were isolated from 9.2%, but *Listeria monocytogenes *from 20.0% of feed samples. Most often different feed concentrates (9.3%) and silage (4.7%) were contaminated with *Listeria monocytogenes*. *Listeria* genus species were isolated more often from feed prepared and used in organic dairy farm than from the feed used in conventional dairy farms, correspondingly 44.4% and 18.3%.

No* Listeria monocytogenes* were found in bulk milk samples from organic dairy farm (*n* = 33), but they were found three times in samples of conventional dairy farms (*n* = 211).

## Figures and Tables

**Table 1:** Incidence of *Listeria* species in feed of dairy cows.

Type of feed	*Listeria *spp. *n* (%)	*L. monocytogenes n* (%)
Grass (*n* = 14)	Not detected	Not detected
Silage (*n* = 48)	4 (3.1)	6 (4.7)
Haylage (*n* = 16)	2 (1.5)	2 (1.5)
Hay (*n* = 10)	Not detected	2 (1.5)
Feed concentrates (*n* = 32)	Not detected	12 (9.3)
Mixed fodder prepared in the farm (*n* = 4)	2 (1.5)	2 (1.5)
Straw ( *n* = 6)	4 (3.1)	2 (1.5)
Total (*n* = 130)	12 (9.2)	26 (20.0)

**Table 2:** Share of* Listeria *spp. and *Listeria monocytogenes *in feed depending of type of farm and season.

Season	Organic farming			Conventional farming		
	Number of samples	*Listeria *spp., *n* (%)	*L. monocytogenes, n* (%)	Number of samples	*Listeria * spp., *n* (%)	*L. monocytogenes, n* (%)
Winter	19	4 (7.4)	10 (18.5)	30	2 (2.6)	8 (10.5)
Spring	14	2 (3.7)	2 (3.7)	14	0	0
Summer	6	0	0	14	0	0
Autumn	15	2 (3.7)	4 (7.4)	18	2 (2.6)	2 (2.6)
Total	54	8 (14.8)	16 (29.6)	76	4 (5.2)	10 (13.1)
